# Linking TB and the Environment: An Overlooked Mitigation Strategy

**DOI:** 10.1289/ehp.116-a478

**Published:** 2008-11

**Authors:** Charles W. Schmidt

One of the world’s leading killers, tuberculosis (TB) is nearly as old as humanity itself; fossilized evidence of this lethal infection has been found in a *Homo erectus* skeleton half a million years old. But look to a modern hospital in a developed country, and you’d be hard pressed to find more than a few cases. Historically, TB is a disease of the poor. Most of the 2–3 million people who die of the disease every year come from the developing world or from poor, urban neighborhoods in wealthier nations. For centuries, TB has been linked anecdotally with environmental risk factors that go hand-in-hand with poverty: indoor air pollution, tobacco smoke, malnutrition, overcrowded living conditions, and excessive alcohol use. Now scientists are presenting convincing evidence to back these associations, leading some TB experts to argue that control programs must confront underlying risk factors to limit the spread of the disease.

According to the World Health Organization (WHO), more than 9 million new cases of TB are diagnosed annually, 55% of them in Asia and 31% in Africa, placing TB second only to HIV/AIDS in terms of the global burden of infectious disease. TB control strategies adopted by the WHO and other organizations emphasize clinical solutions in the form of drugs, vaccines, and access to health care. But despite the success of these programs, TB incidence and mortality aren’t falling rapidly enough to meet WHO targets, and in some areas, particularly in parts of Asia and throughout sub-Saharan Africa, they continue to climb. “Better treatment is essential, but if we want to affect longer-term trends in the epidemic we will also have to deal with risk factors,” asserts Eva Rehfuess, a scientist with the WHO Department of Public Health and Environment. “But doing that won’t be easy. Social and environmental interventions aren’t usually delivered or funded by the health sector, so that means we all have to work with other sectors, in particular housing, energy, and education, to move them forward.”

Emerging evidence suggests such interventions could yield big benefits. In a report published online 3 October 2008 ahead of print in *The Lancet*, Majid Ezzati and colleagues at the Harvard School of Public Health predict that by 2033 TB incidence rates in parts of China could be 14–52% lower if tobacco smoking and indoor air pollution from traditional cooking stoves are eliminated. These reductions assume that 80% of the population already has access to optimal treatments for TB; the benefits would be even greater among those without such access, says Ezzati, an associate professor of global health and environmental health.

## The Current Situation

One-third of the world’s population is thought to be infected with various strains of *Mycobacterium tuberculosis*, the microbe that causes TB, but only 5–10% of infected individuals will develop active disease. Among those with latent infections, which are nontransmissible, *M. tuberculosis* exists in a mysterious dormant state. Precisely why the bacterium favors latency is unknown. Immunity appears to play a role, given that dormant TB often converts to active disease when immune systems falter, for instance, as a consequence of HIV/AIDS. But some TB strains might also favor latency as an evolved trait that fosters survival, says Christopher Dye, an epidemiologist in the WHO Stop TB Department and coauthor of “Expanding the Global Tuberculosis Control Paradigm: The Role of TB Risk Factors and Social Determinants,” a chapter in the forthcoming WHO report *Priority Public Health Conditions: From Learning to Action on Social Determinants of Health*. “It’s possible that latency evolved as a mechanism to avoid extinction when human populations were small and isolated,” he explains. “There are lots of ideas, but our understanding of the bacteria is primitive, and we haven’t come close to explaining the basic facts yet.”

TB drugs are a two-edged sword, says Tommie Victor, a professor of health sciences at Stellenbosch University in South Africa’s Western Cape Province. They destroy the bacterium, but can also select for resistant bacteria against which those drugs are then ineffective. In the 1970s, the drugs had the upper hand, and TB seemed to be in decline. But funding and interest in TB control programs also declined, says Victor, and for the following 20 years no systematic monitoring of drug resistance was carried out. The situation changed dramatically with the arrival of HIV/AIDS in the 1980s, when transmission of TB and outbreaks of multidrug-resistant (MDR) TB increased around the globe.

Drug-susceptible TB cases can be cured with a standardized 6-month regimen of first-line antibiotics administered using the “directly observed treatment, short-course,” or DOTS, protocol. Under the DOTS protocol, patients take their daily medication in the presence of a health care worker to ensure compliance. Considered one of the most effective clinical programs ever, DOTS is a cornerstone of the WHO Stop TB Strategy, which by 2015 aims to reduce the incidence of new TB cases to 50% of 1990 levels. (The long-term goal is to reduce annual TB incidence to fewer than 1 case per 1 million people by 2050.)

When TB patients don’t comply with treatment, or when they take their drugs intermittently, resistant bacteria survive preferentially while drug-susceptible strains die. Thus, noncompliant patients diagnosed initially with drug-susceptible TB can “acquire” MDR TB over time. MDR TB now accounts for 1 of every 20 new cases, making the global TB epidemic a far more urgent problem.

Studies by the Boston-based Partners In Health, a nonprofit supplier of health care services to developing countries, have shown MDR TB treatments can achieve cure rates of up to 80%. But MDR TB patients who remain noncompliant with treatment can acquire a still more worrisome form of TB—dubbed extensively drug-resistant (XDR) TB—that resists practically every known drug at doctors’ disposal.

This lethal disease, discovered for the first time in 2005 in South Africa’s KwaZulu-Natal Province, has a far worse prognosis. This is especially true for patients coinfected with HIV/AIDS, which blunts the immune response and severely exacerbates the downward spiral for those sickened by TB. A study published in the 7 August 2008 *New England Journal of Medicine* found that among 48 XDR TB patients in Peru, daily supervised treatment at community control centers achieved cure rates of 60%. However, none of these patients were HIV-positive. In comparison, all but 1 of 53 HIV-infected individuals stricken with XDR TB in KwaZulu-Natal Province in 2005 and 2006 died within several weeks of developing TB symptoms.

Other researchers have produced particularly dire assessments of XDR TB’s lethality. Citing results from his survey of 4,000 TB cases across several European countries, published in the May 2007 issue of *Emerging Infectious Diseases*, Giovanni Battista Migliori, director of the WHO Collaborating Centre for TB and Lung Diseases, says XDR TB’s mortality rate is up to 5 times greater than that of MDR TB.

It was previously thought that XDR TB, once acquired, might not be infectious beyond hospitals and other clinical settings. XDR TB bacteria were believed to be too weak to be broadly transmissible, presumably because the bacteria are so mutated that they’re generally unhealthy.

Scientists have begun questioning that assumption. Ted Cohen, an epidemiologist and assistant professor at Harvard Medical School, believes some drug-resistant mutations might not exert the so-called fitness costs that would otherwise compromise XDR TB bacteria and weaken them. XDR TB strains that harbor these rare mutations, he predicts, might survive preferentially and eventually predominate over time, especially if more effective drug treatments against these strains remain elusive. Should fit strains prevail, a nightmare scenario might follow: aggressive and transmissible XDR TB spreading inexorably through human populations.

Given the growing global threat of TB, the health sector is working hard to find patients with active disease and get them on a treatment program quickly before they spread the disease. Indeed, active case finding and improved access to health care drive the TB agenda at organizations and institutions such as the WHO and the National Institute of Allergy and Infectious Diseases.

But evidence shows that even more than clinical advances, environmental health improvements have driven historical declines in TB prevalence. In 1979, Thomas McKeown, a professor of social medicine at the University of Birmingham, United Kingdom, wrote in his book *The Role of Medicine* that annual TB death rates throughout England and Wales had declined steadily since at least 1838, when they approached nearly 400 per 100,000.

By the time antibiotics and a TB vaccine first became widely available in the 1940s, decreased crowding, better housing, and improved sanitation and nutrition had pushed TB incidence in England and Wales to fewer than 50 deaths per 100,000, McKeown’s analysis shows. At that point, clinical advances both augmented and accelerated rate declines toward their current levels. And in many parts of the developed world, TB rates still decline today. As of 2006, the United Kingdom’s annual TB incidence was 15 cases per 100,000 (with 935 total deaths), according to the WHO, while the annual U.S. rate was 4 per 100,000 (with 1,310 total deaths).

But where poverty lurks and the influence of environmental risk factors grows (including parts of some developed nations), TB rates soar. This is particularly true in Africa, where HIV/AIDS is pervasive, and in Eastern Europe, where health services have broken down following the collapse of the Soviet Union, the WHO reports in *Global Tuberculosis Control 2008: Surveillance, Planning, Financing*. In Russia’s overcrowded prisons—populated largely by young, poor people, many of whom drink heavily—TB incidence leapt toward 5,000 cases per 100,000 during the late 1990s and early 2000s, with mortality approaching 40% at certain points, among the highest rates in the world, according to Salmaan Keshavjee, an assistant professor at Harvard Medical School. Annual TB incidence in the rural districts surrounding Cape Town, South Africa, where much of the population lives in shantytowns, exceeds 1,300 cases per 100,000, says Victor. South Africa ranks fourth on the WHO’s list of countries with the greatest TB burden, just behind India, China, and Indonesia.

“Our TB patients are practically living on top of each other,” observes Andreas Diacon, a pulmonologist based at Tygerberg Hospital, outside Stellenbosch. “And many of them are malnourished, HIV-positive, and prone to alcohol abuse. Not only does crowding make it more likely that you’ll be exposed to TB, but substance abuse also weakens your immune system. We have statistically more drinkers in both our MDR and XDR populations, and these people are also less likely to stick with medication.”

## Confronting Environmental Risk Factors

Those who argue in favor of risk factor interventions say these strategies offer broad population-level benefits. Consider smoking cessation, says Donald Enarson, senior advisor to the International Union Against Tuberculosis and Lung Disease, an independent research and outreach group in Paris. Although its capacity to trigger TB among individuals is surely less than that of HIV/AIDS, smoking might actually trigger more TB cases on a population basis because people who smoke far outnumber those infected with HIV, he says.

Dye and colleagues at the WHO Stop TB Department, including medical officer Knut Lönnroth, have conducted population-level studies of risk factors. According to their analyses, malnutrition, indoor air pollution from solid fuel use, and active smoking constitute the three top population-attributable TB risks globally, followed by HIV infection, diabetes, and excessive drinking. The results will be published in “Expanding the Global Tuberculosis Control Paradigm.”

When considering these factors, it’s important to distinguish infection risks (i.e., situations that bring people who have TB in close contact with others) from those that accelerate disease progression among people who are already infected. For 40 years, crowding has been cited as a crucial infection threat in both industrialized and nonindustrialized countries. But this factor’s unique contribution to TB risk is hard to quantify, concedes Lönnroth. “The relative risks [from crowding] vary with housing quality, TB prevalence in the community, and also with access to health care, which is associated with chance of early cure of infectious cases,” he says.

Recent findings on crowding and TB infection risk were published by Michael Baker and colleagues at the He Kainga Oranga/Housing and Health Research Programme, University of Otago, New Zealand, in the August 2008 *Journal of Epidemiology and Community Health*. The study revealed that among those aged 40 years or younger, every 1% increase in the proportion of overcrowded households in a given census block led to an 8% increase in TB incidence in that block, holding other variables constant.

Those results support more general findings from India, which show that compared with rural areas, TB prevalence rates are consistently higher among urban children aged 9 years or younger. Vineet Chadha, a senior epidemiologist at the National Tuberculosis Institute in Bangalore, who published those findings in the May 2004 issue of the *International Journal of Tuberculosis and Lung Disease*, says two factors explain the urban/rural discrepancy: first, slum conditions facilitate TB transmission; and second, many urban health practitioners don’t adhere to the DOTS protocol for TB detection and treatment.

The WHO agrees that poverty and urbanization create the perfect conditions for TB transmission. Urbanization leads to higher population densities, crowded living conditions, and increased mobility among migrants seeking temporary work.

A report on crowding and TB, created by researchers at the University of Otago under contract with the WHO, is expected by the end of the year, Rehfuess says. “My sense is that crowding is an important risk factor, but the exposure measures used in epidemiological studies so far haven’t been very reliable,” she says. “So we cannot claim to have conclusive evidence for a causal effect yet.” She points out that many studies show an association—the issue is whether the association is causal or not, as crowding tends to be associated with many other poor socioeconomic and living conditions that could confound the observed relationship.

The links between cigarette smoking and TB, on the other hand, are well documented. Worldwide approximately 1.1 billion people smoke cigarettes, including 930 million in developing or middle-income countries, according to the WHO. At the same time, half the world’s population cooks on open fires or traditional stoves fueled by coal or biomass (wood, animal dung, crop residues, or charcoal), often indoors in poorly ventilated spaces. These stoves produce smoke with chemical constituents—for example, carbon monoxide—similar to those in cigarette smoke. Rehfuess points out that in the developing world most women do not smoke, and most men do not cook for their families. Therefore, in these settings, smoking is one of the greatest TB risk factors for men, whereas indoor air pollution from solid fuel use is likely to be one of the greatest for women. However, a meta-analysis published by Ezzati’s doctoral student Hsien-Ho Lin in the January 2007 *PLoS Medicine* suggests exposure to secondhand tobacco smoke—not just active smoking—also elevates TB risk, making cigarette smoke exposure a risk factor for both sexes.

Since the 1940s, epidemiologists have tried to isolate the unique contributions of tobacco and biomass smoke to TB risk, but early studies did not control adequately for confounders including socioeconomic status, nutrition, alcohol use, housing, and crowding. A turning point came in 2003, when Vendhan Gajalakshmi, a scientist with the Epidemiological Research Center in Chennai, India, and colleagues published a large retrospective investigation that concluded half the TB mortality among Indian men is triggered by active tobacco smoking. This case–control study of 43,000 male deaths and 35,000 controls was published in the 16 August 2003 issue of *The Lancet* and sparked a flurry of new investigations.

Lin’s meta-analysis in the January 2007 *PLoS Medicine* and another by epidemiology professor Michael Bates in the 26 February 2007 *Archives of Internal Medicine* helped to confirm that smoke and TB are causally linked. By combining data from dozens of existing studies, meta-analyses reinforce shared conclusions, Ezzati explains. “The findings of any single study could be produced by chance, or it could show an effect whose magnitude is smaller or larger than the true one,” he says. “But when you combine many studies that look at the same question and they all point in the same general direction, then you can have more confidence that the conclusion you’re getting is correct.”

Although proposed links between smoke exposure and TB might be more defensible now, the underlying mechanisms remain unclear, Ezzati concedes. According to Lin, some scientists speculate that cigarette smoke boosts TB infection risk by impairing the ability of lung cilia to clear bacteria from the respiratory tract.

But exposure might also raise progression risk, adds Adrie Steyn, an assistant professor of microbiology at The University of Alabama at Birmingham, by flooding the body with carbon monoxide. This gas, produced naturally by the body, helps regulate a type of programmed cell death called apoptosis. Evidence suggests apoptosis might account for TB latency. But under high carbon monoxide exposure conditions, such as those induced by cigarette smoking and indoor burning of biomass, apoptosis dramatically declines, Steyn says. And that, he concludes, might allow TB-infected cells to survive and flourish.

More than a matter of medical intrigue, the effects of smoking on the infection/progression continuum pose a real concern for the public health response, Lin says. “DOTS treatment acts on decreasing the probability of infection in the population,” he explains. “If you find that smoking acts more on progression than infection, then DOTS treatment and smoking cessation might complement each other. But if smoking is related more to infection, then the beneficial effect of combining DOTS with smoking cessation is smaller.”

Excessive alcohol use, meanwhile, appears to raise progression and infection risks alike, the former by impairing immune responses, and the latter by inviting risky social interactions that foster transmission of the disease. Smoking and drinking tend to go hand-in-hand, which makes it hard to tease out the effect of one from the other, says Lönnroth. He is lead author of a new meta-analysis of 3 cohort and 18 case–control studies, produced by the WHO Stop TB Department and published 14 August 2008 in *BMC Public Health*, that strives to isolate alcohol’s role in TB. According to that analysis, TB risk is elevated among people who consume more than 40 grams of alcohol per day (the amount found in roughly three 12-oz glasses of beer, 5-oz glasses of wine, or 1.5-oz shots of hard liquor). “Smoking was controlled for in many but not all of the studies in our review, and when we separated out the studies that controlled for smoking, the relative risk [associated with alcohol] tended to be even higher,” Lönnroth says. “The bottom line is that both are independent risk factors, and together they might be particularly strong risk factors.”

On a population basis, malnutrition ranks as the risk factor most commonly linked with TB, according to the forthcoming “Expanding the Global Tuberculosis Control Paradigm.” Still, “the evidence [for that association] in humans is surprisingly thin from the perspective of scientific rigor,” write J. Peter Cegielski and David N. McMurray in a review of human and animal studies published in the March 2004 issue of the *International Journal of Tuberculosis and Lung Disease*. Citing evidence from observations in humans, work in experimental animals, and inferences from related studies in other fields, the authors note that a “wealth of ecological associations link TB with malnutrition in populations affected by famine, war, natural disasters, poverty, mass migration, and confinement in prisons or ghettos.”

Still, human studies are problematic, they concede, because TB itself causes wasting, immune system depression, and other symptoms resembling malnutrition. Given that it’s usually impossible to retrospectively discern nutritional status in patients before their TB disease, it’s hard to determine whether malnutrition led to TB or vice versa. This can only be done reliably through cohort studies that follow healthy subjects over time after determining nutrition status at baseline, says Lönnroth.

Studies from animal models focus on the role of micronutrients, such as proteins and vitamins. The results of such studies, Cegielski and McMurray claim, suggest that protein deficiency in particular impedes both innate and vaccine-induced resistance to TB, although precisely how it does so remains unclear. The two authors conclude that in the aggregate, evidence suggests that malnutrition and TB are related; therefore, “nutritional support of undernourished populations at high risk of TB may reduce the incidence of TB in such groups.”

Keshavjee agrees with that conclusion. “When you’re not eating well, you’re probably not producing collagen and immune proteins properly, and that leads to problems with lung parenchyma [i.e., the integrity of the lung tissue itself] and also with immune function,” he says. “We could be talking about something as simple as vitamin D deficiency. Vitamin D helps with macrophage function, and macrophages help to clear TB bacteria. So if you don’t have enough [vitamin D], you could be at higher risk.”

Adding to a link with nutrition is growing evidence that diabetes exacerbates TB risk. A meta-analysis by Christie Jeon and Megan Murray, published 15 July 2008 in *PLoS Medicine*, concluded “diabetes is associated with TB risk regardless of study design and population.” Indeed, says Enarson, with an increase in high-fat/low-nutrition diets with and lack of exercise, the combination of diabetes and smoking will probably cause more TB worldwide in the next 2 to 3 years than any other factor.

## Applying Risk Factors to TB Control

With accumulating evidence showing that environmental factors exacerbate TB risk, health experts are contemplating how to incorporate these findings into TB control programs. For a start, says Rehfuess, health professionals should inform patients and their families of the potential harm resulting from excessive drinking, smoking, indoor biomass burning, and other practices. “Raising awareness and creating demand for solutions will not solve the problem,” she says, “but it could be a stepping stone. And even that is not happening.”

Murray and others suggest that high-risk populations, including smokers and diabetic patients, might be targeted for active case finding. Locating patients with TB remains a difficult challenge. The disease can take weeks to diagnose; meanwhile, untreated patients pose infection risks to their communities. Looking within high-risk populations might make those efforts more efficient. Moreover, tying case detection to enticements such as improved cooking stoves might also make for good strategy, Ezzati proposes. “Those who agree to be tested for TB regularly, along with those who agree to quit smoking, might be entitled to a clean stove benefit,” Ezzati says. “And that would have benefits beyond TB, because these pollutants also increase risks for other lung diseases.”

Partners In Health has already incorporated risk factors into their treatment approach, Keshavjee says. The organization supports community treatment programs and helps families pay to build an additional room to house the infected patient, thereby reducing the risk of crowding, while also supplying clean fuels such as kerosene to reduce indoor air pollution. Keshavjee concedes these measures come at a cost to the organization, but he emphasizes the downstream expenses posed by inadequately treated TB are far higher.

Thus far, the WHO hasn’t integrated risk factors into its TB control efforts. The problem, Dye points out, is that changes in the risk factor trends that have traditionally accompanied TB fluctuations occur slowly—over decades or even centuries. “Smoke exposure and other factors heighten TB risk, but it’s difficult to imagine how we could manipulate these things to bring down TB incidence quickly,” he says. “It would be beneficial if these risk factors did-n’t exist, but they’re not going to replace prompt drug treatment, which is still the most powerful way to save lives quickly and reduce transmission. Risk factor interventions are useful, but they’re supplementary.”

Still, the authors of “Expanding the Global Tuberculosis Control Paradigm” acknowledge that “even if the Stop TB strategy is successfully implemented . . . the global incidence rate by 2050 would be about 100 times greater than the elimination target. Interventions to reduce progression to disease may include preventive treatment with anti-TB drugs, a new vaccine that prevents progression from infection to disease, as well as reducing exposure to various social, environmental, and biological risk factors for TB.” Adds Keshavjee, “[Environmental] risk factor controls have not typically been under the purview of the TB community. To address them, we have to think outside the box.”

## Figures and Tables

**Figure:**
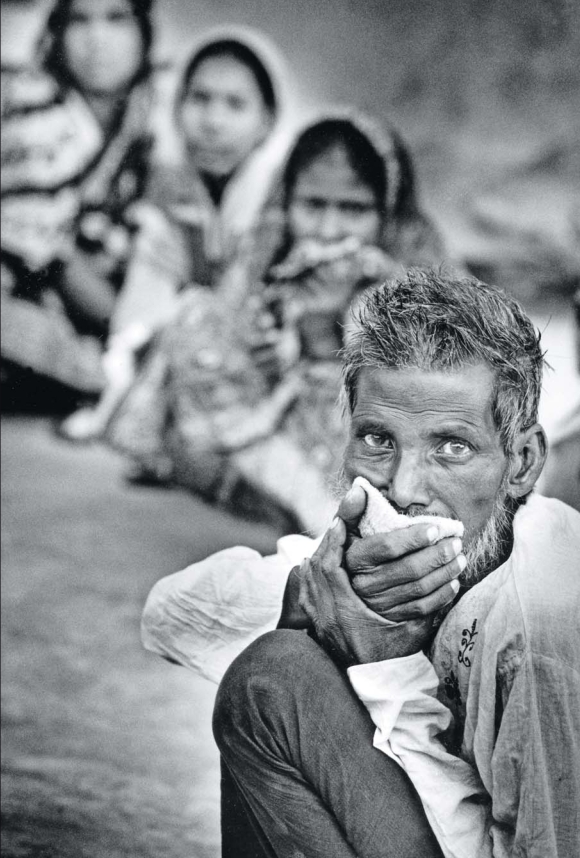
A man suffering from TB arrives at a hospital in Mymensingh, Bangladesh. Bangladesh has the sixth greatest TB burden in the world.

**Figure:**
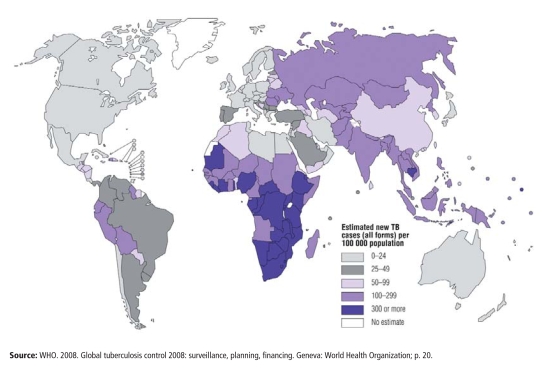
Estimated Incidence of New TB Cases as of 2006

**Figure:**
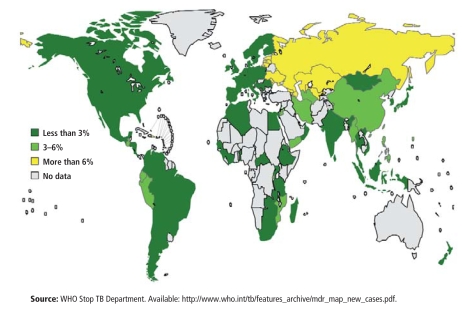
Percentage of MDR TB among New TB Cases, 1994 – 2007

**Figure:**
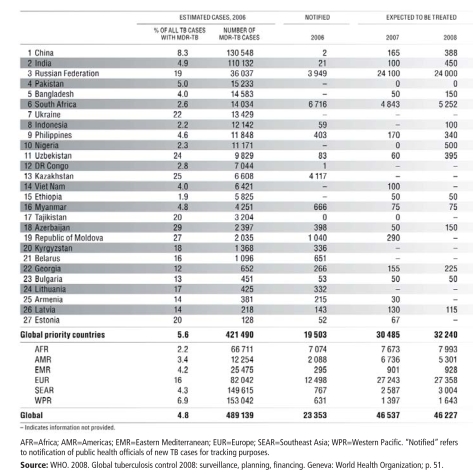
Countries with the Most MDR TB among New TB Cases

**Figure:**
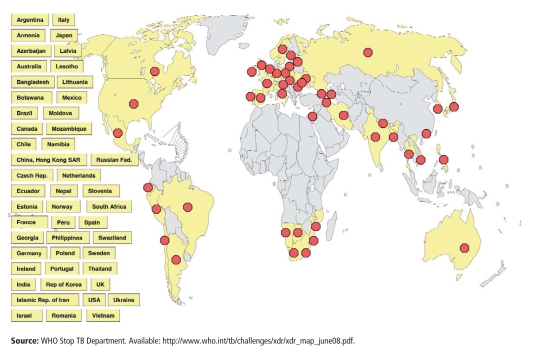
Countries with Confirmed Cases of XDR TB as of June 2008

**Figure:**
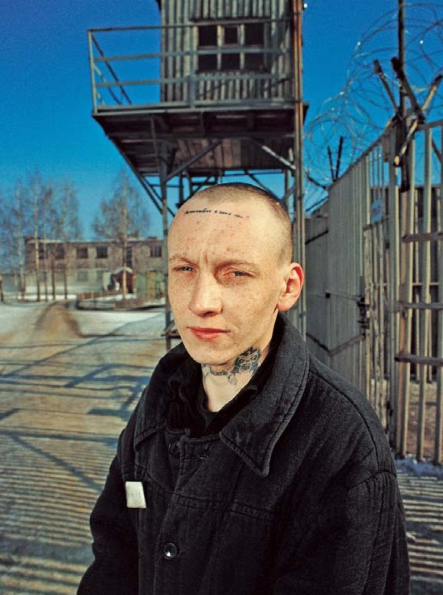
Arkadiy Yusev, age 23, is a Russian prisoner and TB patient. The disease is rampant in the overcrowded prisons of the Russian Federation, which has the eleventh greatest TB burden in the world.

**Figure:**
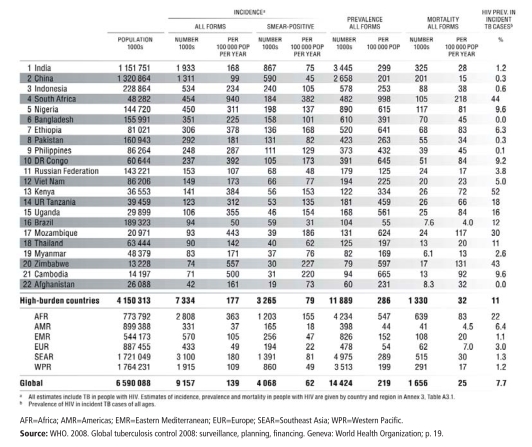
Countries with the Greatest TB Burden as of 2006

**Figure:**
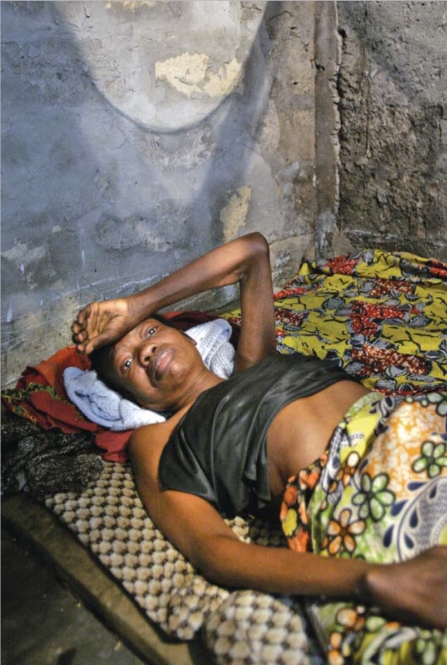
For many TB patients, such as this terminally ill woman in Kinshasa, DR Congo, effective drugs are prohibitively expensive. DR Congo has the tenth greatest TB burden in the world.

